# Insights into the Molecular Mechanisms of Purine Compounds Synergistically Inducing Larval Settlement in *Mytilopsis sallei* Using Multi-Group Comparative Transcriptomic Analysis

**DOI:** 10.3390/biology13121067

**Published:** 2024-12-20

**Authors:** Jian He, Huanhuan Hao, Huakang Pan, Shanshan Yao, Yiran Zhao, Shifeng Guo, Jianfang Huang, Danqing Feng

**Affiliations:** 1 Fuzhou Institute of Oceanography, Minjiang University, Fuzhou 350108, China; hejian@mju.edu.cn; 2 College of Ocean & Earth Sciences, Xiamen University, Xiamen 361102, China; hhhao@stu.xmu.edu.cn (H.H.); 22320221151378@stu.xmu.edu.cn (H.P.); 22320220156394@stu.xmu.edu.cn (S.Y.); zhaoyiran@stu.xmu.edu.cn (Y.Z.); 3 Shenzhen Key Laboratory of Smart Sensing and Intelligent Systems, Shenzhen Institute of Advanced Technology, Chinese Academy of Sciences, Shenzhen 518055, China; sf.guo@siat.ac.cn

**Keywords:** larval settlement, transcriptome, *Mytilopsis sallei*, purine, AMPK

## Abstract

The settlement of marine benthic invertebrate larvae is a crucial stage in their life cycle, directly influencing their population distribution, growth, and reproduction. It is generally believed that multiple chemical inducers in the environment synergistically affect larval settlement, though the underlying molecular mechanisms remain unclear. Our previous study has established that three purine compounds (adenosine, inosine, and hypoxanthine) released by adults of the mussel *Mytilopsis sallei* synergistically induced larval settlement. In this study, we performed transcriptomic sequencing analysis on larvae exposed to individual purine compounds and those exposed to a combination of three purine compounds. A total of 21,850 differentially expressed genes (DEGs) were identified. KEGG enrichment analysis of these DEGs revealed that the AMPK signaling pathway, FoxO signaling pathway, extracellular matrix (ECM)-receptor interaction, focal adhesion, and the thyroid hormone signaling pathway play key roles in the settlement of *M. sallei* larvae. Moreover, in larvae exposed to the combined action of three purines, the expression of relevant signaling pathways and metabolic processes was more significant. This research provides important information for understanding the molecular mechanisms underlying the induction of larval settlement in *M. sallei* by purine compounds in natural environments.

## Simple Summary

The life cycle of marine benthic invertebrates includes a planktonic larval stage and a benthic adult stage, with larval settlement being the critical transition from a planktonic to a benthic lifestyle. Since adult mobility is limited or completely lost in the benthic environment, larval settlement directly affects population distribution, growth, and reproduction. Although larval settlement induced by natural chemical cues is widespread among marine invertebrates, the molecular mechanisms underlying this process remain unclear. *Mytilopsis sallei*, a typical marine fouling mussel, releases three purine compounds—adenosine, inosine, and hypoxanthine—that synergistically induce larval settlement. In this study, transcriptomic sequencing was employed to identify differentially expressed genes and signaling pathways associated with purine-compound-induced larval settlement. This research not only provides new insights into the molecular mechanisms of larval settlement in marine benthic invertebrates, but also offers a scientific basis and theoretical foundation for the ecological control of marine fouling organisms.

## 1. Introduction

Most benthic marine invertebrates exhibit a characteristic biphasic life cycle, consisting of a planktonic larval stage followed by a benthic adult stage [1]. Following the planktonic phase, larvae sink onto the substrate, engage in substrate exploration, and undergo settlement [2]. Subsequently, they undergo metamorphosis into juveniles and further develop into adults. Given the limited or entirely absent motility of benthic adults, larval settlement and metamorphosis play a crucial role in determining the spatial distribution, population dynamics, and community structure of benthic marine invertebrates [3]. As planktonic larvae of benthic marine invertebrates develop, they acquire settlement and metamorphic competence, during which they can perceive exogenous inductive signals, with environmental chemical cues being identified as critical determinants in initiating these processes [4]. Subsequently, under the modulation of endogenous regulatory pathways, larval tissues and organs undergo catabolic remodeling, while new anatomical structures are synthesized, culminating in the completion of metamorphosis.

In natural environments, multiple environmental chemical cues are typically involved in the induction of settlement and metamorphosis in marine invertebrate larvae. For example, Franco et al. [5] isolated three bacterial strains, namely *Shigella flexneri*, *Microbacterium liquefaciens*, and *Kocuria erythromyxa*, from the natural substrates associated with the hydroid *Hydractinia symbiolongicarpus,* which were capable of synthesizing acyl-homoserine lactones (AHLs). Their findings indicate that the combined effects of crude extracts containing these bacterial quorum-sensing molecules can synergistically promote larval settlement in hydroids. In addition, Guo et al. [6] demonstrate that two bacterium-derived metabolites, (lyso)phospholipids and curdlan, can synergistically induce larval metamorphosis in the hydroid *H. echinata*, which may help ensure optimal habitat selection. Despite the increasing body of literature demonstrating that natural chemical cues work synergistically to induce larval settlement and metamorphosis in marine invertebrates, the mechanisms underlying this synergistic interaction remain inadequately elucidated.

Studying the mechanisms by which chemical compounds synergistically influence larval settlement in marine invertebrates presents numerous challenges. Firstly, larval settlement is a complex biological process regulated by various internal and external factors, which may interact in non-linear ways, complicating experimental design and data analysis [7]. Secondly, the synergistic effects of multiple compounds likely involve the co-regulation of several signaling pathways, making it difficult for any single method to fully elucidate these mechanisms. Additionally, accurately simulating and controlling factors such as the concentration, combination, and duration of chemical inducers in the marine environment is particularly challenging. In recent years, advancements in sequencing technologies have facilitated the application of transcriptomic and proteomic approaches to elucidate the mechanisms of synergistic interactions among various compounds [8]. Despite the limitations of transcriptomic approaches in this context, they offer the advantage of high-throughput identification of differentially expressed genes and signaling pathways related to settlement. Transcriptomics allows us to map out the response profiles of larvae to chemical compounds, offering a promising step forward in the field and providing new perspectives for uncovering the molecular mechanisms underlying the synergistic induction of larval settlement.

Our previous study has established that three purine compounds (adenosine, inosine, and hypoxanthine) released by adults of the mussel *Mytilopsis sallei* synergistically induced larval settlement and metamorphosis [9]. Furthermore, we demonstrated that adenosine promoted larval settlement and metamorphosis via the adenosine kinase–AMP-activated protein kinase–Forkhead box O signaling pathway [10]. However, the synergistic mechanisms underlying the interactions of these three purine compounds remain poorly understood. This study performs transcriptomic analyses on larval settlement induced by adenosine, inosine, hypoxanthine, and the combination of these three purines. Differentially expressed genes and signaling pathways are identified, and the critical regulatory signaling pathways involved in the synergistic induction of larval settlement by the purine compounds are examined. This research provides important information for understanding the molecular mechanisms underlying the induction of larval settlement in *M. sallei* by purine compounds in natural environments.

## 2. Materials and Methods

### 2.1. Larval Culture of M. sallei

*M. sallei* adults (shell length, 20–30 mm) were collected from the Yundang Lagoon, Xiamen, China (24°48′ N, 118°09′ E). Spawning induction and larval culture were carried out in the laboratory following our published protocol [11]. After 6–8 days of incubation, larvae were able to swim in the seawater and crawl on the substrate with their foot for short intervals, indicating that they had reached the pediveliger stage. Pediveligers with an average shell length of 232.8 ± 37.1 μm, capable of settlement and metamorphosis, were selected for bioassays and transcriptomic analyses.

### 2.2. Bioassays of Larval Settlement and Metamorphosis

Bioassays were conducted in sterile six-well polystyrene Petri plates following the previous study [12]. Three replicates were set up for each treatment. In each replicate, 30–40 pediveliger larvae were added to each well containing 10 mL of a test solution. Filtered seawater (FSW) was used as a control. The six-well Petri plates were maintained at 27 °C in the dark. After 48 h of incubation, larval settlement and metamorphosis were observed through a Leica inverted microscope (DM IL LED). Larval settlement was identified by crawling on the substrate using a foot or by attaching with byssus, while metamorphosis was confirmed by the loss of the velum or the appearance of mature gill filaments.

### 2.3. Sample Collection for Transcriptomic ANALYSIS and qRT-PCR Analysis

Sample collection was conducted as in our previous study [10]. Briefly, pediveligers of *M. sallei* were collected and transferred into five tanks (45 × 35 × 25 cm). In these tanks, 10 L of FSW was added, ensuring a larval density of about 5 larvae/mL. Four tanks were supplemented with adenosine (final concentration 1.25 μM), inosine (final concentration 1.25 μM), hypoxanthine (final concentration 3.75 μM), and a mixture of the three purines (final concentration 6.25 μM), respectively, while the other tank served as the blank control with no added chemicals. After 24 h of incubation, a large number of larvae had attached to the bottom of the tanks. At this point, planktonic larvae from the control tank were collected into three cryovials, frozen in liquid nitrogen, and labeled as P (pediveliger larvae). Attached larvae were gently brushed into filtered seawater using a soft brush, collected into cryovials, and rapidly frozen in liquid nitrogen after the removal of excess seawater. These samples were labeled as NS (naturally settled larvae), AS (adenosine-induced settled larvae), IS (inosine-induced settled larvae), HS (hypoxanthine-induced settled larvae), and MS (mixture-induced settled larvae), respectively.

### 2.4. Gene Expression Validation by qRT-PCR

The qRT-PCR experiment was conducted following the SYBR^®^ Premix Ex Taq™ kit (Takara Biotechnology (Dalian) Co., Ltd., Dalian, China) protocol. Based on the transcriptome library of *M. sallei* larvae, the ORF sequences of AMPK, FoxO, PEPCK, FasL, TRAIL, and ATG8 genes were obtained. Primers for qRT-PCR for each gene were designed using Primer Premier 5 software (Table S1). The qRT-PCR analysis was carried out following our published protocol [10]. The relative gene expression levels were calculated using the 2^−ΔΔCt^ method with β-actin as the internal control.

Details of total RNA extraction, transcriptomic library construction, and sequencing are provided in the Supplementary Materials.

### 2.5. Statistical Analysis

The results of the bioassay and qRT-PCR were analyzed with SPSS 22.0 software. One-way analysis of variance (ANOVA) was performed with a Dunnett’s post hoc test for multiple comparisons of treatment means with the control.

## 3. Results

### 3.1. Synergistic Effects of Purines on Larval Settlement and Metamorphosis in M. sallei

Our previous study identified that three purine compounds (adenosine, inosine, and hypoxanthine) released by adult *M. sallei* synergistically induced larval settlement and metamorphosis in a ratio of 1:1:3, with their minimum effective concentrations being 0.01 μM, 0.01 μM, and 0.03 μM, respectively [9]. At these concentrations, the individual compounds did not elicit any inducing effects. To elucidate the mechanisms of synergistic effects through transcriptomic analyses, it is critical to determine optimal concentrations at which the individual compounds can effectively induce larval settlement and metamorphosis, while retaining their inducing effects when combined. This study establishes baseline concentrations of 0.01 μM adenosine (Ado), 0.01 μM inosine (Ino), and 0.03 μM hypoxanthine (Hyp), subsequently increasing these concentrations proportionally to evaluate the effects of both individual and combined treatments on larval settlement and metamorphosis of *M. sallei*.

**Figure 1:**
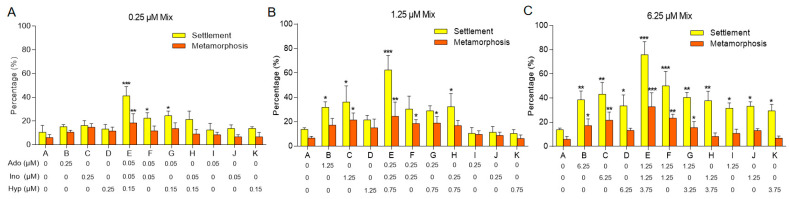
Effects of various concentrations of purines on larval settlement and metamorphosis of *M. sallei*. (**A**) Percentage of larval settlement and metamorphosis in response to 0.25 μM of purine mixtures. (**B**) Percentage of larval settlement and metamorphosis in response to 1.25 μM of purine mixtures. (**C**) Percentage of larval settlement and metamorphosis in response to 6.25 μM of purine mixtures. 0.25 μM Mix, 1.25 μM Mix, 6.25 μM Mix: the sum of concentrations of the mixtures were 0.25 μM, 1.25 μM and 6.25 μM, respectively. Asterisks denote a significant difference compared with the control (* *p* < 0.05, ** *p* < 0.01, *** *p* < 0.001, Dunnett’s test).

At a total concentration of 0.25 μM, both the combination of all three compounds (treatment E) and combinations of two compounds (treatments F, G, H) significantly enhanced larval settlement compared to the control (treatment A), while individual treatments at 0.25 μM (treatments B, C, D) exhibited no significant effects on settlement or metamorphosis (Figure 1A).

When the total concentration was raised to 1.25 μM, individual treatments of Ado and Ino at 1.25 μM (treatments B, C) also resulted in significant induction of larval settlement. However, other treatments, including the 1.25 μM Hyp treatment alone (treatment D) and the individual treatments of 0.25 μM Ado, Ino, and Hyp (treatment I, J, K), showed no significant effects on larval settlement or metamorphosis (Figure 1B).

At a total concentration of 6.25 μM, all treatment groups, including individual treatments of Ado, Ino, and Hyp (treatments I, J, K), significantly enhanced larval settlement. Furthermore, it was evident that the percentages of settlement and metamorphosis were markedly higher in the three-compound mixture compared to other treatments, illustrating a synergistic effect (Figure 1C).

**Figure 2:**
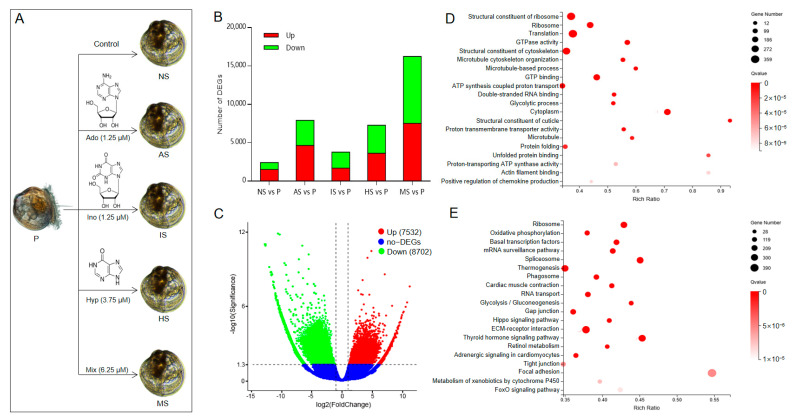
Identification and enrichment analysis of differentially expressed genes. (**A**) The samples collected for transcriptome analysis. Ado: adenosine; AS: adenosine-induced settled larvae; Ino: inosine; IS: inosine-induced settled larvae; HS: hypoxanthine-induced settled larvae; Hyp: hypoxanthine; MS: mixture-induced settled larvae; NS: naturally settled larvae; P: pediveliger larvae. (**B**) Number of differentially expressed genes between settled larvae and pediveliger larvae. (**C**) Volcano plots of differentially expressed genes between mixture-induced settled larvae and pediveliger larvae. Red and green spots indicate the upregulated expressed genes and the downregulated expressed genes between settled larvae and pediveliger larvae, respectively. Blue spots indicate genes that were not differentially expressed. (**D**) Bubble plots of GO enrichment analysis of the differentially expressed genes between mixture-induced settled larvae and pediveliger larvae. (**E**) Bubble plots of KEGG enrichment analysis of the differentially expressed genes between mixture-induced settled larvae and pediveliger larvae.

Based on these results, to further elucidate the molecular mechanisms underlying the synergistic induction of larval settlement and metamorphosis in *M. sallei* by purine compounds, the concentrations of adenosine, inosine, and hypoxanthine were set at 1.25 μM, 1.25 μM, and 3.75 μM, respectively (Figure 2A).

### 3.2. Transcriptome Sequencing, Unigene Annotation, and Classification of Unigenes

A summary of sequencing data quality is shown in Table S2. After filtering, the number of non-redundant sequences (clean reads) ranged from 42,456,470 to 46,105,012, accounting for 92.62% to 94.55% of the raw reads. The sequencing quality of the 18 samples used for assembly, as measured by Q20 and Q30 (both quality standards for assessing read accuracy), exceeded 96% and 88%, respectively. Overall, the transcriptome sequencing results were of high quality, meeting the requirements for subsequent assembly and analysis.

A total of 306,189 Unigenes were identified and subjected to BLASTx analysis against seven public databases (NR, NT, KO, Swiss-Prot, PFAM, GO, and KOG). Through bioinformatics annotation, 150,167 Unigenes were successfully annotated (Table 1), representing 42.77% of the total genes. Of these, 87,807 Unigenes (38.19%) were annotated in the NR database, 42,233 Unigenes (28.18%) in the Swiss-Prot database, with annotation rates of 23.60% and 25.58% for the GO and KOG databases, respectively.

According to the NR annotation results, the species distribution of annotated Unigenes is presented in Figure S1A. Among the 57,355 Unigenes annotated in the NR database, 13,657 (23.82%) were annotated to the scallop *Mizuhopecten yessoensis*, 8990 (15.68%) to the oyster *Crassostrea gigas*, and 7683 (13.4%) to the oyster *Crassostrea virginica*. Additionally, a significant proportion of genes (37.98%) were annotated to other species. A total of 35,439 Unigenes were annotated to the GO database, covering the three main GO categories: Biological Process (BP), Cellular Component (CC), and Molecular Function (MF). These Unigenes were mapped to 44 GO terms (Figure S1B).

Following the KO annotation of 45,359 genes, they were categorized based on their involvement in KEGG metabolic pathways, as shown in Figure S1C. Notably, the category with the highest representation was Signal Transduction, comprising 8070 genes (17.79%). A total of 38,406 Unigene sequences were annotated in the KOG database, yielding classification information for gene homologs. KOG is divided into 25 functional groups, as shown in Figure S1D. The categories with the highest number of associated genes were “General Function Prediction” and “Signal Transduction Mechanisms”, with 7356 and 5655 genes, respectively.

### 3.3. Identification and Enrichment Analysis of Differentially Expressed Genes

Based on the gene expression profiles across different samples, the significantly differentially expressed genes (DEGs) were identified, as illustrated in Figure 2B. In the comparison between naturally settled larvae and pediveliger larvae (NS vs. P), 2406 DEGs were detected, with 1514 genes upregulated and 892 downregulated. For adenosine-induced settled larvae vs. pediveliger larvae (AS vs. P), 7912 DEGs were identified, including 4642 upregulated and 3270 downregulated genes. In the inosine-induced settled larvae vs. pediveliger larvae (IS vs. P) comparison, 3772 DEGs were observed, with 1696 upregulated and 2076 downregulated. Hypoxanthine-induced settled larvae vs. pediveliger larvae (HS vs. P) revealed 7282 DEGs, with 3618 upregulated and 3664 downregulated. Lastly, in the purine-mixture-induced settled larvae vs. pediveliger larvae (MS vs. P) comparison, 16,234 DEGs were identified, with 7532 upregulated and 8702 downregulated (Figure 2C). These findings suggest a marked increase in DEG numbers following the treatment with the purine compound mixture.

The GO enrichment analysis results showed that in the NS vs. P group, 689 DEGs were significantly enriched in 55 GO terms, with 465 DEGs highly significantly enriched in 21 GO terms, including chitin metabolic process, extracellular matrix, and protein folding (Table S3). In the AS vs. P group, 1844 DEGs were significantly enriched in 37 GO terms, with 1234 DEGs highly significantly enriched in 29 GO terms, including protein folding, actin filament binding, and sarcomere (Table S4). In the IS vs. P group, 731 DEGs were significantly enriched in 48 GO terms, with 982 DEGs highly significantly enriched in 28 GO terms, such as ribosome structural components, translation, protein folding, and GTP binding (Table S5). In the HS vs. P group, 2912 DEGs were significantly enriched in 78 GO terms, with 2171 DEGs highly significantly enriched in 36 GO terms, including ribosome structural components, translation, ribosomes, and cytoskeletal structural components (Table S6). In the MS vs. P group, 3536 DEGs were significantly enriched in 87 GO terms, with 2832 DEGs highly significantly enriched in 43 GO terms, including ribosome structural components, translation, ribosomes, and GTPase activity (Table S7, Figure 2D). These results indicate that the number of GO terms enriched with DEGs significantly increased after the combined treatment of the three purine compounds.

Further analysis identified the GO terms that were enriched across all groups (Table 2). The results revealed 14 significantly enriched GO terms, including chitin metabolic process, protein folding, and dopamine metabolic process. In the MS vs. P group, most GO terms showed an increasing trend in the number of DEGs, except for synaptic transmission, dopamine metabolic process, and neuron cellular homeostasis. The chitin metabolic process, chitin binding, and cuticle structure composition are likely involved in the formation of the juvenile shell [13]. The dopamine metabolic process and synaptic transmission may play roles in neural signal transduction [14], while the regulation of gated potassium ion channels could be related to membrane depolarization [15]. A previous study has confirmed that both dopamine and potassium ions can induce larval settlement and metamorphosis in *M. sallei* [12], suggesting that these GO terms play crucial roles in regulating the settlement and metamorphosis of *M. sallei* larvae.

The KEGG enrichment analysis revealed that in the NS vs. P group, 758 DEGs were significantly enriched in 10 pathways, including extracellular-matrix-receptor interaction, focal adhesion, and the PI3K-Akt signaling pathway (Table S8). In the AS vs. P group, 2429 DEGs were significantly enriched in 12 pathways, including extracellular-matrix-receptor interaction, the PI3K-Akt signaling pathway, and protein digestion and absorption (Table S9). In the IS vs. P group, 1019 DEGs were significantly enriched in 11 pathways, including the ribosome, dorsoventral axis formation, and Th1 and Th2 cell differentiation (Table S10). In the HS vs. P group, 3698 DEGs were significantly enriched in 23 pathways, including the ribosome, oxidative phosphorylation, and basal transcription factors (Table S11). In the MS vs. P group, 5153 DEGs were significantly enriched in 30 pathways, including the ribosome, oxidative phosphorylation, and basal transcription factors (Table S12, Figure 2E). It is evident that after the combined treatment of the three purine compounds, the number of DEGs enriched in KEGG pathways significantly increased.

Further analysis filtered the KEGG pathways enriched across all groups (Table 3). The results indicated that four KEGG pathways were significantly enriched, namely extracellular-matrix-receptor interaction, focal adhesion, the PI3K-Akt signaling pathway, and the thyroid hormone signaling pathway. Notably, the number of DEGs in these pathways significantly increased in the MS vs. P group. It is hypothesized that these pathways play a crucial role in regulating the settlement and metamorphosis of *M. sallei* larvae.

### 3.4. Expression Analysis of Key Genes in the AMPK-FoxO Signaling Pathway

**Figure 3:**
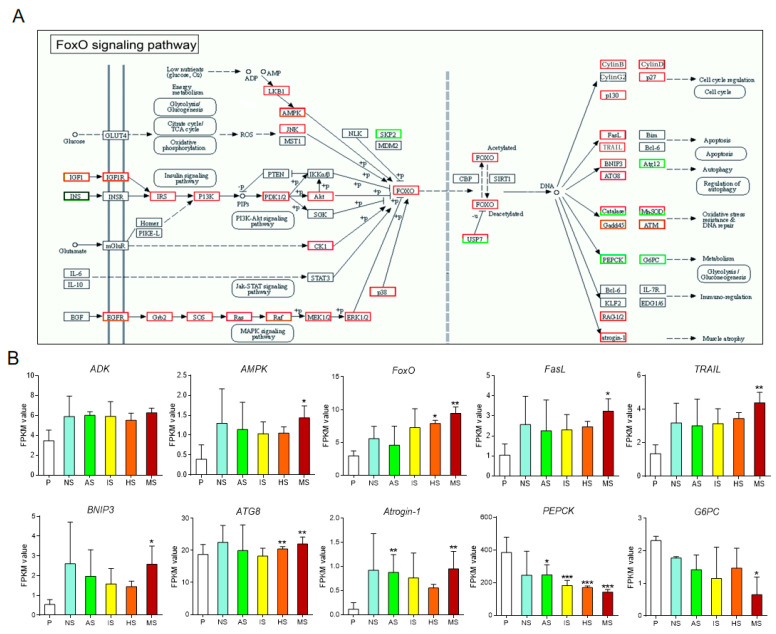
Expression analysis of key genes in the AMPK-FoxO signaling pathway. (**A**) FoxO signaling pathway constructed based on KEGG pathway analysis for mixture-induced settled larvae vs. pediveliger larvae. Red and green boxes represent the genes that were upregulated and downregulated, respectively. Genes shown in boxes lined with both red and green are annotated by different contigs, which may be either upregulated (red) or downregulated (green). (**B**) Expression levels of key genes from the ADK-AMPK-FoxO signaling pathway. AS: adenosine-induced settled larvae; IS: inosine-induced settled larvae; HS: hypoxanthine-induced settled larvae; MS: mixture-induced settled larvae; NS: naturally settled larvae; P: pediveliger larvae. Asterisks denote a significant difference compared with the pediveliger larvae (* *p* < 0.05, ** *p* < 0.01, *** *p* < 0.001).

Previous research identified that adenosine-induced larval settlement in *M. sallei* is mediated by adenosine kinase (ADK), which catalyzes the reaction between adenosine and ATP to produce AMP and ADP, subsequently activating the AMPK-FoxO signaling pathway [10]. In the present study, KEGG enrichment analysis revealed that the FoxO signaling pathway was significantly enriched in both the HS vs. P group (Table S11) and MS vs. P group (Table S12, Figure 3A), while the AMPK signaling pathway was only significantly enriched in the MS vs. P group (Table S12). To confirm that the synergistic effects of the purine compounds indeed influence the AMPK-FoxO signaling pathway, the expression of key genes in this pathway was further analyzed. Transcriptomic data indicate that the expression of the ADK gene exhibited no significant differences before and after natural settlement, nor following settlement induced by individual or combined purine compounds (Figure 3B). In contrast, AMPK gene expression was significantly elevated in the mixture-induced settled larvae compared to the pediveliger larvae (*p* = 0.032), whereas individual purine compounds did not notably alter AMPK expression levels (Figure 3B). Regarding the FoxO gene, its expression was significantly upregulated following hypoxanthine-induced settlement (*p* = 0.014), with no significant changes observed after adenosine or inosine treatment. Notably, the combined application of all three purine compounds led to a more pronounced upregulation of FoxO expression compared to the control (*p* = 0.0012).

The AMPK-FoxO signaling pathway is known to regulate cell apoptosis and autophagy [16,17]. In this study, we observed that the expression of apoptosis-related genes, tumor necrosis factor FasL and TRAIL, was significantly upregulated (*p* = 0.018 and *p* = 0.004, respectively) following the combined induction by three purines, while individual purine treatments did not result in significant expression changes (Figure 3B). Regarding autophagy regulation, the expression of autophagy-related genes BNIP3 and ATG8 was significantly upregulated after the synergistic action of the purines (*p* = 0.011 and *p* = 0.0014, respectively). In contrast, BNIP3 showed no significant expression changes after individual purine treatments, whereas ATG8 expression was significantly upregulated following hypoxanthine treatment alone (*p* = 0.0025). In addition, the muscle autophagy gene Atrogin-1 was significantly upregulated under the combined action of the purines (*p* = 0.002), and its expression was also significantly increased following adenosine treatment alone (*p* = 0.003).

Additionally, the AMPK-FoxO signaling pathway also regulates the glycolysis/gluconeogenesis pathways [18]. This study found that after the combined action of three purines, the expression level of the key enzyme glucose-6-phosphatase (G6PC) in the gluconeogenesis pathway was significantly downregulated (*p* = 0.034), whereas no significant differential expression was observed with individual purine treatment. Moreover, each of the three purine compounds alone significantly inhibited the expression of another key enzyme, phosphoenolpyruvate carboxykinase (PEPCK), with the lowest expression level observed under the combined action of the three compounds (*p* = 0.0002).

Overall, after natural settlement of *M. sallei* larvae, key genes in the AMPK-FoxO signaling pathway exhibited differential expression compared to pre-settlement, but the changes were not significant. When a single purine compound was used for induction, a few key genes in the signaling pathway showed significant differential expression. However, under the combined action of three purine compounds, a greater number of key genes in the signaling pathway exhibited significant differential expression, with the effects being more pronounced compared to the action of individual compounds.

**Figure 4:**
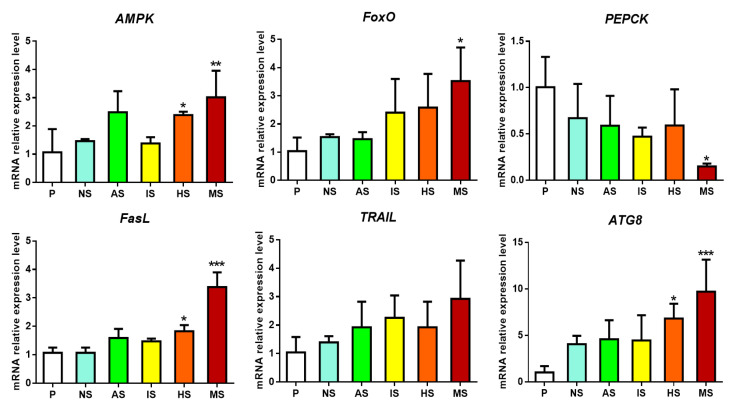
mRNA relative expression levels of key genes from AMPK-FoxO signaling pathway analyzed by qRT-PCR. AS: adenosine-induced settled larvae; IS: inosine-induced settled larvae; HS: hypoxanthine-induced settled larvae; MS: mixture-induced settled larvae; NS: naturally settled larvae; P: pediveliger larvae. Asterisks denote a significant difference compared with the pediveliger larvae (* *p* < 0.05, ** *p* < 0.01, *** *p* < 0.001).

Furthermore, the validation of key genes’ expression in the AMPK-FoxO signaling pathway was conducted using qRT-PCR. The results demonstrated that when induced with a single purine compound, genes in the AMPK signaling pathway exhibited differential expression. In contrast, after the combined application of three purines, more genes showed significant differential expression, and the effects were more pronounced compared to the individual compound treatments (Figure 4). This finding aligns closely with the quantitative results from RNA-Seq (Figure 3), confirming the reliability of the transcriptomic analysis.

### 3.5. Expression Analysis of Genes Related to Byssus Secretion Proteins

Transcriptome analysis identified multiple genes associated with byssus secretion, including foot protein genes, adhesive plaque matrix protein genes, and byssal calumenin-like protein genes. The expression patterns of these genes were evaluated to understand their roles in larval settlement under the influence of purine compounds.

**Figure 5:**
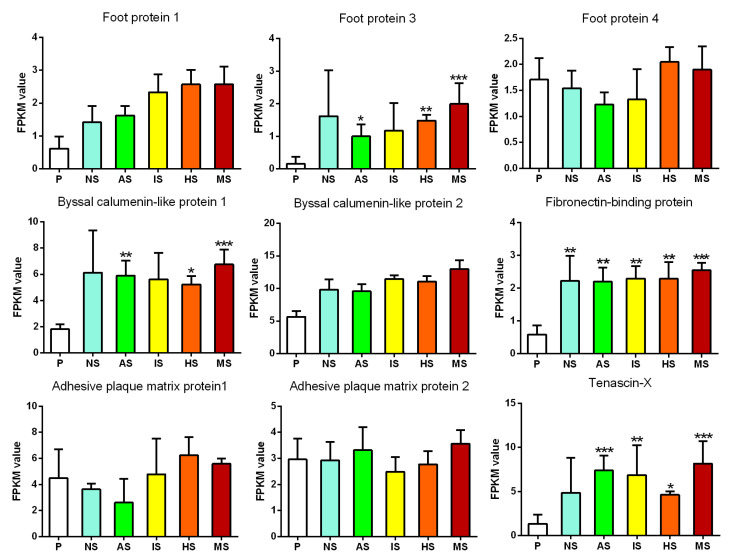
Expression analysis of genes related to byssus secretion proteins. AS: adenosine-induced settled larvae; IS: inosine-induced settled larvae; HS: hypoxanthine-induced settled larvae; MS: mixture-induced settled larvae; NS: naturally settled larvae; P: pediveliger larvae. Asterisks denote a significant difference compared with the pediveliger larvae (* *p* < 0.05, ** *p* < 0.01, *** *p* < 0.001).

A total of three foot protein genes (foot protein 1, 3, 4) were identified. The expression levels of foot protein 1 and foot protein 4 showed no significant differences before and after larval settlement. However, the foot protein 3 was significantly upregulated after induction by adenosine and hypoxanthine (*p* = 0.048 and *p* = 0.006, respectively), and its expression was more dramatically upregulated under the combined action of purine compounds (*p* < 0.001) (Figure 5).

Two adhesive plaque matrix proteins were identified, and neither showed significant differential expression before and after larval settlement. Additionally, two byssal calumenin-like proteins were identified. The expression of calumenin-like protein 1 was significantly upregulated under the influence of adenosine and hypoxanthine and highly significantly upregulated under the combined action of purine compounds (*p* < 0.001), while calumenin-like protein 2 showed no significant expression changes before and after settlement (Figure 5).

The fibronectin-binding protein gene was significantly upregulated after natural settlement (*p* = 0.034), with even more pronounced upregulation under the induction of individual purine compounds, and its expression was highly significantly upregulated under the combined effect of the three purine compounds (*p* < 0.001, Figure 5). The Tenascin-X gene showed an increase in expression after natural attachment, though not significant (*p* = 0.07). However, it was significantly upregulated after individual treatment with adenosine, inosine, and hypoxanthine (*p* = 0.0007, 0.005, and 0.036, respectively), with even more pronounced upregulation under the combined action of the three purine compounds (*p* = 0.00032, Figure 5).

Overall, after natural settlement of *M. sallei* larvae, genes related to byssus secretion exhibited differential expression compared to pre-settlement, though not significantly. When a single purine compound was applied, some byssus-secretion-related genes showed significant differential expression. However, under the combined effect of the three purines, more genes showed significant differential expression, with the effects being more pronounced than under the action of individual compounds.

## 4. Discussion

In this study, KEGG enrichment analysis revealed that neither Ado nor Ino alone significantly affected the AMPK or FoxO signaling pathways. However, when Hyp was applied individually, it significantly impacted the FoxO signaling pathway, leading to the differential expression of 76 genes (Table S11). When all three purine compounds were combined, both the AMPK and FoxO signaling pathways were significantly affected, with 102 and 94 DEGs identified, respectively (Table S12). These results suggest that the synergistic induction of larval settlement in *M. sallei* by the three purine compounds may be regulated through the AMPK-FoxO signaling pathway, further confirming the critical role of this pathway in regulating the settlement and metamorphosis of *M. sallei* larvae [10].

AMPK plays a crucial regulatory role in various metabolic pathways, particularly in maintaining intracellular energy homeostasis [19,20]. Studies have shown that the activation of both the AMPK and FoxO signaling pathways can enhance glycolysis or inhibit gluconeogenesis [21,22]. This study also identified significant enrichment of the glycolysis/gluconeogenesis pathway (ko00010) in the HS vs. P and MS vs. P groups, with 51 and 56 differentially expressed genes (DEGs) identified, respectively. Further quantitative analysis showed that phosphoenolpyruvate carboxykinase (PEPCK)—a key rate-limiting enzyme in the gluconeogenesis pathway responsible for catalyzing the conversion of oxaloacetate to phosphoenolpyruvate—was significantly reduced after purine compound treatment, with the lowest expression observed in the combined treatment group (Figure 3B and Figure 4). Since larval metamorphosis in marine invertebrates is an energy-intensive process requiring energy reserves before metamorphosis [23,24], and glycolysis is an energy-producing process while gluconeogenesis is an energy-consuming process, it is hypothesized that the induction of larval settlement by purine compounds promotes glycolysis or inhibits gluconeogenesis, thereby providing energy reserves necessary for larval metamorphosis.

Apoptosis has been demonstrated to play a pivotal regulatory role in the settlement and metamorphosis of marine invertebrate larvae [25]. In this study, it was observed that apoptosis-related genes downstream of the AMPK-FoxO signaling pathway, including tumor necrosis factors FasL and TRAIL, showed upregulated expression after co-induction by the three purine compounds, whereas no significant expression changes were detected when the compounds were applied individually (Figure 3B). Furthermore, autophagy-related genes, such as BNIP3 and ATG8, along with the muscle-specific autophagy gene Atrogin-1, also exhibited significantly increased expression following combined purine compound induction (Figure 3B). It is hypothesized that these genes may play an essential regulatory role in the degradation of specialized larval tissues, such as the velum. The co-induction of purine compounds appears to significantly upregulate these apoptosis- and autophagy-related genes, potentially facilitating tissue remodeling during larval settlement and metamorphosis of *M. sallei*.

Extracellular matrix (ECM)-related proteins are known to directly or indirectly regulate a variety of cellular activities, including cell migration, adhesion, differentiation, proliferation, and apoptosis [26]. Focal adhesions are dense structures formed on the cell membrane when integrins cluster locally and recruit and phosphorylate a series of intracellular proteins after binding to their corresponding ligands in the ECM [27]. Recent studies have shown that shell growth in marine bivalves involves several cellular processes, including the participation of ECM-receptor and focal-adhesion-related proteins. For instance, Zhang et al. [28] found that in the proteome of the oyster *Crassostrea gigas* larvae, in addition to RNA-transport-related proteins, ECM-receptor proteins were among the most abundant shell matrix proteins, with a significant presence of focal adhesion proteins as well. Similarly, in the shell growth of the oyster *C. virginica*, large amounts of ECM and focal adhesion proteins were identified [29], suggesting that these proteins play critical roles in the regulation of shell biomineralization. In this study, both the ECM-receptor interaction pathway (ko04512) and the focal adhesion signaling pathway (ko04510) were significantly enriched following natural settlement and compound-induced settlement, with even stronger enrichment observed under the combined effect of the three purine compounds. This suggests that ECM-receptor interactions and focal adhesion signaling pathways are likely involved in regulating the metamorphosis of *M. sallei* larvae, particularly in the formation of the juvenile shell during this process.

In vertebrates, thyroid hormones (THs) have been confirmed to regulate various physiological processes, including hormone-mediated developmental stages [30]. Amphibian metamorphosis, for instance, is heavily dependent on THs, which induces a range of physiological changes such as morphogenesis, cell death, and tissue remodeling [31]. In fish, THs have been shown to induce the metamorphosis of the Japanese eel *Anguilla japonica* from the leptocephalus larval stage to the juvenile stage, also through thyroid hormone receptor (TR)-mediated mechanisms [32]. Recent studies have extended these findings to marine invertebrates, suggesting a role for TR in the regulation of larval metamorphosis. For example, Wang et al. [33] found that triiodothyronine (T3) significantly induced metamorphosis in the larvae of the abalone *Haliotis diversicolor* and successfully cloned the thyroid hormone receptor gene (HdTR). RNA interference (RNAi) targeting HdTR reduced the T3-induced metamorphic effect, indicating the involvement of TR in the regulation of metamorphosis. In this study, the thyroid hormone signaling pathway (ko04919) was significantly enriched following both natural settlement and compound-induced settlement, with more pronounced enrichment observed under the combined effect of the three purine compounds. This suggests that the thyroid hormone signaling pathway is involved in regulating the larval settlement process of *M. sallei*. However, the detailed mechanisms underlying this involvement require further investigation.

The larval settlement of *M. sallei* larvae is closely related to byssus secretion, which is a complex process involving a series of biochemical reactions [34]. Li et al. (2017) [35] sequenced the genome of the scallop *Chlamys farreri* and identified 16 byssus-related proteins. Functional annotation revealed that these proteins include tyrosinases and peroxidases (involved in redox reactions), the ECM protein tenascin-X (which promotes ECM coagulation), and serine protease inhibitors and metalloproteinase inhibitors (which prevent biodegradation). To further investigate the molecular mechanisms of byssus secretion in scallops, the authors performed transcriptomic sequencing on different parts of the foot (proximal, middle, distal) at various time points of byssus secretion. The results showed that the proximal part of the foot (near the byssus gland) significantly overexpressed tenascin-X. In this study, we found that the expression of the tenascin-X gene was upregulated after natural settlement, though not significantly. However, after individual treatment with adenosine, inosine, and hypoxanthine, the expression was significantly upregulated, and the upregulation was even more pronounced under the combined action of the three purine compounds (*p* = 0.00032, Figure 5). This result further suggests that tenascin-X plays an important role in byssus secretion in mollusks and may also promote the hardening of the byssus in *M. sallei*.

## 5. Conclusions

This study identified a total of 21,850 differentially expressed genes (DEGs), with 2406 DEGs in the NS vs. P group, 11,912 DEGs in the AS vs. P group, 3772 DEGs in the IS vs. P group, 11,282 DEGs in the HS vs. P group, and 16,234 DEGs in the MS vs. P group. KEGG enrichment analysis of these DEGs revealed that the AMPK signaling pathway, the FoxO signaling pathway, the glycolysis metabolic pathway, ECM-receptor interaction, focal adhesion, and the thyroid hormone signaling pathway play key roles in the settlement of *M. sallei* larvae. Moreover, in the MS group, the expression of relevant signaling pathways and metabolic processes was more significant. These findings provide valuable insights into the molecular mechanisms underlying the purine-compound-induced settlement of *M. sallei* larvae in natural environments.

## Figures and Tables

**Table 1:** Summary of Unigene annotation against seven databases.

Database	Number of Genes	Percentage (%)
Annotated in NR	57,355	38.19
Annotated in NT	14,755	9.83
Annotated in KO	45,359	30.21
Annotated in SwissProt	42,322	28.18
Annotated in PFAM	46,174	30.75
Annotated in GO	35,439	23.60
Annotated in KOG	38,406	25.58
Annotated in all databases	7479	4.98
Annotated in at least one database	64,227	42.77
Total Unigenes	150,167	100

**Table 2:** Significantly enriched GO terms with DEG numbers across all groups.

GO Term	NS vs. P	AS vs. P	IS vs. P	HS vs. P	MS vs. P
Biological Process					
Chitin metabolic process	47	98	98	72	88
Protein folding	21	47	30	51	56
Caveola assembly	13	16	14	20	21
Synaptic transmission, dopaminergic	3	3	3	3	3
Dopamine metabolic process	3	3	3	3	3
Neuron cellular homeostasis	3	3	3	3	3
Positive regulation of voltage-gated potassium channel activity	9	12	10	19	22
Cellular Component					
Caveola	13	6	14	20	21
Molecular Function					
Chitin binding	47	101	93	73	89
Metalloendopeptidase activity	30	60	35	66	87
Unfolded protein binding	17	41	24	46	49
Calcium ion binding	97	319	221	275	392
GPI anchor binding	9	12	10	19	22
Structural constituent of cuticle	7	5	10	22	25

**Table 3:** Significantly enriched KEGG pathways with DEG numbers across all groups.

Pathway Name	NS vs. P	AS vs. P	IS vs. P	HS vs. P	MS vs. P
ECM-receptor interaction	95	223	178	254	282
Focal adhesion	128	315	120	295	400
PI3K-Akt signaling pathway	107	270	105	229	317
Thyroid hormone signaling pathway	78	260	114	256	335

## Data Availability

Data pertaining to this work are available from the corresponding authors upon reasonable request.

## Supplementary Materials

The following supporting information can be downloaded at https://www.mdpi.com/article/10.3390/biology13121067/s1: Supplementary Methods: total RNA extraction and quality assessment, transcriptome library construction and sequencing; transcriptome assembly and functional annotation, gene expression level analysis, differential expression gene analysis, GO and KEGG enrichment analysis [36,37,38,39,40]; Figure S1: Unigene Classification; Table S1: Primers for qRT-PCR; Table S2: Sequencing data quality summary; Table S3: Significantly enriched GO terms of DEGs in NS vs. P group; Table S4: Significantly enriched GO terms of DEGs in AS vs. P group; Table S5: Significantly enriched GO terms of DEGs in IS vs. P group; Table S6: Significantly enriched GO terms of DEGs in HS vs. P group; Table S7: Significantly enriched GO terms of DEGs in MS vs. P group; Table S8: Significantly enriched KEGG pathways of DEGs in NS vs. P group; Table S9: Significantly enriched KEGG pathways of DEGs in AS vs. P group; Table S10: Significantly enriched KEGG pathways of DEGs in IS vs. P group; Table S11: Significantly enriched KEGG pathways of DEGs in HS vs. P group; Table S12. Significantly enriched KEGG pathways of DEGs in MS vs. P group.
