# Supplementary material for: Insights into the Molecular Mechanisms of Purine Compounds Synergistically Inducing Larval Settlement in Mytilopsis sallei Using Multi-Group Comparative Transcriptomic Analysis
Source: Biology (Basel). 2024 Dec 20;13(12):1067. doi: 10.3390/biology13121067 (PMC11672916; doi:10.3390/biology13121067)
Supplement: Supplementary file 1 [file biology-13-01067-s001.zip › biology-3316339-supplementary.pdf]

Supplementary Materials

Article

Insights into the Molecular Mechanisms of Purine Compounds Synergistically Inducing Larval Settlement in *Mytilopsis sallei* Using Multi-Group Comparative Transcriptomic Analysis

Jian He [1], Huanhuan Hao [2], Huakang Pan [2], Shanshan Yao [2], Yiran Zhao [2], Shifeng Guo [3], Jianfang Huang [1,*] and Danqing Feng [2,*]

[1] Fuzhou Institute of Oceanography, Minjiang University, Fuzhou 350108, China; hejian@mju.edu.cn
[2] College of Ocean & Earth Sciences, Xiamen University, Xiamen 361102, China; hhhao@stu.xmu.edu.cn (H.H.); 22320221151378@stu.xmu.edu.cn (H.P.); 22320220156394@stu.xmu.edu.cn (S.Y.); zhaoyiran@stu.xmu.edu.cn (Y.Z.)
[3] Shenzhen Key Laboratory of Smart Sensing and Intelligent Systems, Shenzhen Institute of Advanced Technology, Chinese Academy of Sciences, Shenzhen 518055, China; sf.guo@siat.ac.cn
* Correspondence: jianfhuang@mju.edu.cn (J.H.); dqfeng@xmu.edu.cn (D.F.)

Supplementary Methods

Total RNA Extraction and Quality Assessment

Following the method described by Yang et al. (2012) [36], total RNA was extracted from 18 samples (6 experimental groups × 3 biological replicates) using the TRIzol method. The detailed steps are as follows:

(1) For each treatment, 100 mg of sample containing about 3,000 pediveliger larvae was taken and mixed with 1 mL of TRIzol reagent. Three sterilized steel beads were added to each EP tube, and the mixture was shaken at 30 times/s for 7 minutes. The tubes were then left at room temperature for 5-10 minutes.

(2) 0.2 mL of chloroform was added, and the mixture was shaken vigorously for 15 seconds before being left on ice for 2 minutes.

(3) The mixture was centrifuged at 12,000 rpm for 15 minutes at 4 °C. After centrifugation, the liquid separated into layers: the upper colorless aqueous phase, which mainly contained RNA, the middle layer, and the bottom red phenol-chloroform layer, which mainly contained DNA and proteins.

(4) The upper aqueous phase was carefully transferred to a new EP tube, and an equal volume of pre-chilled isopropanol was added. The mixture was inverted gently to mix and then left on ice for 30 minutes or stored at -20 °C for 10 minutes.

(5) The mixture was centrifuged at 12,000 rpm for 10 minutes at 4 °C, and the supernatant was discarded (the tube should have a white RNA pellet or colored RNA pellet at the bottom).

(6) 1 ml of 75% ethanol was added along the wall of the tube to gently wash the pellet. The mixture was centrifuged again at 12,000 rpm for 5 minutes at 4 °C. The supernatant was discarded, and this washing step was repeated.

(7) The remaining supernatant was carefully removed, and the RNA pellet was air-dried in a refrigerated centrifuge at 12,000 rpm for 5 minutes at 4 °C.

(8) The residual liquid was removed using a pipette tip, and the pellet was dried in a clean bench. The RNA was then dissolved in 30-50 μl of DEPC-treated water and stored at -80 °C.

(9) The extracted RNA was quantified using a NanoDrop 2000 spectrophotometer to determine the OD260/280 ratio, assessing RNA concentration and purity. Additionally, RNA quality was evaluated by 1% agarose gel electrophoresis, checking for RNA integrity (where the brightness of the 28S band should be approximately twice that of the 18S band).

Transcriptome Library Construction and Sequencing

Library construction and sequencing were outsourced to BGI (Shenzhen, China). The key steps are as follows:

(1) Magnetic beads with Oligo(dT) were used to enrich polyA-tailed mRNA.

(2) Fragmentation Buffer was added to fragment the mRNA.

(3) The first strand of cDNA was synthesized using mRNA as a template with random hexamer primers. The second strand was synthesized using buffer, dNTPs, RNase H, and DNA polymerase I, followed by purification with AMPure XP beads.

(4) The purified double-stranded cDNA underwent end repair, A-tailing, and adapter ligation. Fragment size selection was performed using AMPure XP beads.

(5) PCR amplification was used to enrich the cDNA library.

After library construction, the concentration and insert size of the library were checked using Qubit 2.0 and Agilent 2100, respectively. Library quantification was done using qRT-PCR to ensure quality. Libraries that passed the quality check were sequenced using the Illumina HiSeq 2500 platform, yielding paired-end reads (2×125 bp).

Transcriptome Assembly and Functional Annotation

The transcriptome assembly process included:

(1) Sequencing Data Filtering: Removal of adapter-containing reads, reads with >10% ambiguous bases (N), and low-quality reads where >50% of bases had Qphred ≤ 20.

(2) Transcriptome Assembly: Trinity v2.4.0 [37] was used to assemble clean reads for the de novo transcriptome of samples without a reference genome.

(3) Redundancy Removal: Redundant sequences were removed using CD-HIT v4.5.4 with a threshold of $c = 0.95$.

(4) Obtaining Unique Protein-Coding Unigenes: EvidentialGene v2013.07.27 [38] was used to retrieve unique protein-coding unigenes, with parameters set to MINCDS:90.

To obtain comprehensive gene function information, Unigene sequences were aligned to Nr, Nt, Pfam, KOG/COG, Swiss-Prot, KEGG, and GO databases using BLAST with an E-value threshold of 1e-5.

Gene Expression Level Analysis

The transcriptome assembled by Trinity was used as the reference. Clean reads from each sample were mapped to this reference. Since *Mytilopsis sallei* lacks a reference genome, a non-reference transcriptome was used, and RSEM software [39] was employed to calculate the read count for each gene and convert it to FPKM. Gene expression levels were analyzed based on the expected number of Fragments Per Kilobase of transcript sequence per Million base pairs sequenced (FPKM).

Differential Expression Gene Analysis

Differential expression gene (DEG) analysis was performed using DESeq[40], with a selection threshold of $padj < 0.05$. Since DEG analysis in RNA-seq involves independent statistical hypothesis testing across a large number of genes, it can lead to an overall increase in false positives. Therefore, during the differential analysis, the p-values obtained from the original hypothesis tests were corrected to address this issue.

GO and KEGG Enrichment Analysis of DEGs

DEGs were functionally classified based on the GO annotation and official classifications, and enrichment analysis was performed using the 'phyper' function in R software. P-values were calculated and adjusted for false discovery rate (FDR), with Q-value thresholds of <0.05 and <0.01 for significant and highly significant enrichment, respectively.

For KEGG pathway classification, DEGs were categorized based on KEGG annotations and subjected to enrichment analysis using 'phyper' in R software. Significantly enriched pathways were defined as those with Q-values ≤ 0.05.

Table S1. Primers for qRT-PCR

**Gene ID**	**Gene name**	**Forward primer (5′—3′)**	**Reverse primer (5′—3′)**	**Product size (bp)**
Unigene912_All	β-actin	GTCACGGACGATTTCACGC	CCATCTACGAAGGTTACGCTCT	143
Unigene59823_All	AMPK	AGGAATGGGAGGTTGAGGC	GGGTCTGTGCTTTATTTACTGTTG	207
CL1586.Contig4_All	FoxO	CACAAACAGCAACGAGGGG	AAGCATAGAGCCACCCGAGA	160
CL1684.Contig4_All	PEPCK	CAAAGCGGGTTCGGTTCT	GCCAGCCAGCAGTTCTCAT	163
CL11775.Contig1_All	FasL	ACTCACGAAATGGCAATAAA	CCCTCTTCACGTACACCC	178
CL9734.Contig2_All	TRAIL	CTCGGAAATTAGACTGGGTC	ACTGTGGGTTGGTCGTGT	273
CL7049.Contig2_All	ATG8	TTGATCCGCTTCCTAATGAT	ATACAAAGAAACCCACAAAGAC	198

Table S2. Sequencing data quality summary

Sample ID	Total Raw Reads (M)	Total Clean Reads (M)	Total Clean Bases(Gb)	Clean Reads Q20(%)	Clean Reads Q30(%)	Clean Reads Ratio(%)
P_1	45.57	42.46	6.37	97.25	89.47	93.16
P_2	47.33	43.95	6.59	97.34	89.72	92.87
P_3	47.33	44.26	6.64	97.00	88.88	93.53
NS_1	48.73	45.34	6.80	97.16	89.25	93.03
NS_2	47.33	44.13	6.62	97.31	89.70	93.24
NS_3	45.57	42.21	6.33	97.09	89.22	92.62
AS_1	47.33	44.04	6.61	97.08	89.16	93.06
AS_2	47.33	44.09	6.61	96.81	88.53	93.17
AS_3	47.33	44.08	6.61	97.13	89.11	93.14
IS_1	47.33	44.30	6.64	97.05	89.00	93.60
IS_2	49.08	46.11	6.92	97.21	89.28	93.94
IS_3	47.33	44.30	6.64	97.40	89.81	93.60
HS_1	47.33	43.96	6.59	97.15	89.30	92.88
HS_2	45.57	42.55	6.38	97.14	89.29	93.36
HS_3	49.08	45.79	6.87	97.20	89.49	93.31
MS_1	45.57	42.84	6.43	97.12	89.30	93.99
MS_2	47.33	44.34	6.65	96.89	88.82	93.69
MS_3	45.57	42.89	6.43	97.14	89.32	94.11

Table S3. GO enrichment analysis of the DEGs for NS vs P group

GO Term ID	GO Term	Number of DEGs	Q value
GO:0006030	chitin metabolic process	47	2.53E-09
GO:0008061	chitin binding	47	6.13E-09
GO:0005576	extracellular region	70	3.81E-08
GO:0006457	protein folding	21	1.24E-06
GO:0070836	caveola assembly	13	1.42E-05
GO:0005901	caveola	13	2.36E-05
GO:0004222	metalloendopeptidase activity	30	2.81E-05
GO:0005975	carbohydrate metabolic process	31	5.09E-05
GO:0005452	inorganic anion exchanger activity	8	0.000117838
GO:0051082	unfolded protein binding	17	0.000119956
GO:0001963	synaptic transmission, dopaminergic	3	0.001439055
GO:0008810	cellulase activity	6	0.001439055
GO:0042417	dopamine metabolic process	3	0.001439055
GO:0070050	neuron cellular homeostasis	3	0.001439055
GO:0005509	calcium ion binding	97	0.001694513
GO:0032222	regulation of synaptic transmission, cholinergic	9	0.004554735
GO:1903818	positive regulation of voltage-gated potassium channel activity	9	0.004554735
GO:0034235	GPI anchor binding	9	0.004832885
GO:0042302	structural constituent of cuticle	7	0.005943901
GO:0090114	COPII-coated vesicle budding	3	0.007356512
GO:0000139	Golgi membrane	19	0.009609669

Table S4. GO enrichment analysis of the DEGs for AS vs P group

GO Term ID	GO Term	Number of DEGs	Q value
GO:0006457	protein folding	47	1.91E-05
GO:0051015	actin filament binding	48	1.91E-05
GO:0030017	sarcomere	17	3.70E-05
GO:0005198	structural molecule activity	38	0.000162587
GO:0005885	Arp2/3 protein complex	12	0.000217394
GO:0051082	unfolded protein binding	41	0.000217394
GO:0005882	intermediate filament	17	0.000263697
GO:0004222	metalloendopeptidase activity	30	0.000345436
GO:0005975	carbohydrate metabolic process	84	0.000496166
GO:0006030	chitin metabolic process	98	0.000546167
GO:0008061	chitin binding	101	0.000576776
GO:0070836	caveola assembly	16	0.000666778
GO:0005901	caveola	16	0.000666778
GO:0005576	extracellular region	175	0.000695481
GO:0034314	Arp2/3 complex-mediated actin nucleation	12	0.000771244
GO:0004521	endoribonuclease activity	11	0.001228765
GO:0001963	synaptic transmission, dopaminergic	3	0.001905578
GO:0042417	dopamine metabolic process	3	0.002009055
GO:0070050	neuron cellular homeostasis	3	0.002439945
GO:0034235	GPI anchor binding	12	0.002832885
GO:0008810	cellulase activity	11	0.002028762
GO:0007160	cell-matrix adhesion	29	0.002542499
GO:1903818	positive regulation of voltage-gated potassium channel activity	12	0.004554735
GO:0005509	calcium ion binding	319	0.004694513
GO:0042302	structural constituent of cuticle	5	0.005243901
GO:0090114	COPII-coated vesicle budding	5	0.005246146
GO:0030245	cellulose catabolic process	9	0.007510521
GO:0030247	polysaccharide binding	16	0.007510521
GO:0004553	hydrolase activity, hydrolyzing O-glycosyl compounds	44	0.008657470

Table S5. GO enrichment analysis of the DEGs for IS vs P group

GO Term ID	GO Term	Number of DEGs	Q value
GO:0003735	structural constituent of ribosome	85	1.10E-21
GO:0006412	translation	77	5.77E-18
GO:0005840	ribosome	71	5.43E-17
GO:0006457	protein folding	30	2.06E-09
GO:0051082	unfolded protein binding	24	9.56E-06
GO:0003924	GTPase activity	52	0.000112538
GO:0005525	GTP binding	65	0.000272474
GO:0070836	caveola assembly	14	0.000272474
GO:0002756	MyD88-independent toll-like receptor signaling pathway	5	0.000320788
GO:0005901	caveola	14	0.000320788
GO:0032722	positive regulation of chemokine production	5	0.000320788
GO:0034138	toll-like receptor 3 signaling pathway	5	0.000320788
GO:0043330	response to exogenous dsRNA	5	0.000320788
GO:0006030	chitin metabolic process	98	0.000546167
GO:0008061	chitin binding	93	0.000576776
GO:0022625	cytosolic large ribosomal subunit	13	0.000700603
GO:0004222	metalloendopeptidase activity	35	0.000915877
GO:0042302	structural constituent of cuticle	10	0.000915877
GO:0050729	positive regulation of inflammatory response	5	0.001360773
GO:0005230	extracellular ligand-gated ion channel activity	18	0.001477517
GO:0001963	synaptic transmission, dopaminergic	3	0.004537616
GO:0009396	folic acid-containing compound biosynthetic process	3	0.004537616
GO:0042417	dopamine metabolic process	3	0.004537616
GO:0070050	neuron cellular homeostasis	3	0.004537616
GO:1903818	positive regulation of voltage-gated potassium channel activity	12	0.004655547
GO:0005509	calcium ion binding	221	0.004744513
GO:0034235	GPI anchor binding	10	0.004752885
GO:0030272	5-formyltetrahydrofolate cyclo-ligase activity	3	0.004759903

Table S6. GO enrichment analysis of the DEGs for HS vs P group

GO Term ID	GO Term	Number of DEGs	Q value
GO:0003735	structural constituent of ribosome	198	2.38E-42
GO:0006412	translation	193	2.35E-37
GO:0005840	ribosome	169	1.67E-36
GO:0005200	structural constituent of cytoskeleton	55	2.31E-18
GO:0007017	microtubule-based process	57	3.81E-15
GO:0003924	GTPase activity	148	8.75E-15
GO:0000226	microtubule cytoskeleton organization	38	2.54E-14
GO:0005525	GTP binding	186	2.60E-13
GO:0006457	protein folding	51	2.54E-07
GO:0042302	structural constituent of cuticle	22	2.54E-07
GO:0006096	glycolytic process	20	4.61E-07
GO:0015078	proton transmembrane transporter activity	21	8.72E-07
GO:0051082	unfolded protein binding	46	1.55E-06
GO:0005874	microtubule	68	8.99E-06
GO:0005852	eukaryotic translation initiation factor 3 complex	18	4.51E-05
GO:0015991	ATP hydrolysis coupled proton transport	19	0.001607069
GO:0022627	cytosolic small ribosomal subunit	20	0.001607069
GO:0070836	caveola assembly	20	0.002234374
GO:0005901	caveola	20	0.002320238
GO:0015630	microtubule cytoskeleton	9	0.002362164
GO:2000377	regulation of reactive oxygen species metabolic process	5	0.002445518
GO:0004222	metalloendopeptidase activity	66	0.002515877
GO:1903818	positive regulation of voltage-gated potassium channel activity	19	0.002815547
GO:0022625	cytosolic large ribosomal subunit	21	0.002930186
GO:0045261	proton-transporting ATP synthase complex	7	0.003013608
GO:0006030	chitin metabolic process	72	0.004546167
GO:0008061	chitin binding	73	0.004576776
GO:0034235	GPI anchor binding	19	0.004612885
GO:0019843	rRNA binding	17	0.004615511
GO:0001963	synaptic transmission, dopaminergic	3	0.004637436
GO:0042417	dopamine metabolic process	3	0.004637616
GO:0070050	neuron cellular homeostasis	3	0.004637565
GO:0005509	calcium ion binding	275	0.005244513
GO:0055037	recycling endosome	11	0.005396274
GO:0003723	RNA binding	199	0.008332816

Table S7. GO enrichment analysis of the DEGs for MS vs P group

GO Term ID	GO Term	Number of DEGs	Q value
GO:0003735	structural constituent of ribosome	319	2.58E-34
GO:0005840	ribosome	187	1.21E-28
GO:0006412	translation	313	1.21E-28
GO:0003924	GTPase activity	182	6.57E-15
GO:0005200	structural constituent of cytoskeleton	257	7.02E-14
GO:0000226	microtubule cytoskeleton organization	42	3.34E-13
GO:0007017	microtubule-based process	61	1.04E-11
GO:0005525	GTP binding	224	2.54E-11
GO:0015986	ATP synthesis coupled proton transport	122	9.64E-09
GO:0003725	double-stranded RNA binding	25	1.39E-08
GO:0006096	glycolytic process	24	4.23E-08
GO:0005737	cytoplasm	271	2.40E-07
GO:0042302	structural constituent of cuticle	25	3.28E-07
GO:0015078	proton transmembrane transporter activity	23	6.57E-06
GO:0005874	microtubule	83	3.63E-05
GO:0006457	protein folding	56	4.97E-05
GO:0051082	unfolded protein binding	49	0.000605308
GO:0046933	proton-transporting ATP synthase activity	12	0.000610218
GO:0051015	actin filament binding	53	0.000988638
GO:0032722	positive regulation of chemokine production	7	0.001059168
GO:0034138	toll-like receptor 3 signaling pathway	7	0.001059168
GO:0022627	cytosolic small ribosomal subunit	24	0.002076027
GO:0070836	caveola assembly	21	0.002234374
GO:0005901	caveola	21	0.002320238
GO:1903818	positive regulation of voltage-gated potassium channel activity	22	0.002215547
GO:0004222	metalloendopeptidase activity	87	0.002215877
GO:0003743	translation initiation factor activity	71	0.002215921
GO:0070062	extracellular exosome	12	0.002215921
GO:0001732	formation of cytoplasmic translation initiation complex	22	0.002902962
GO:0043066	negative regulation of apoptotic process	17	0.003318053
GO:0015991	ATP hydrolysis coupled proton transport	22	0.003834315
GO:0001963	synaptic transmission, dopaminergic	3	0.004337436
GO:0042417	dopamine metabolic process	3	0.004537616
GO:0070050	neuron cellular homeostasis	3	0.004537565
GO:0005509	calcium ion binding	392	0.004544513
GO:0015630	microtubule cytoskeleton	10	0.004574827
GO:0008137	NADH dehydrogenase (ubiquinone) activity	14	0.004611566
GO:0006030	chitin metabolic process	88	0.005546167
GO:0008061	chitin binding	89	0.005576776
GO:0034235	GPI anchor binding	22	0.006752885
GO:0055037	recycling endosome	13	0.007539845
GO:0004332	fructose-bisphosphate aldolase activity	8	0.009062765

Table S8. Significantly enriched KEGG pathways of DEGs in NS vs P group

Pathway ID	Pathway Name	Number of DEGs	Q value
ko04512	ECM-receptor interaction	95	6.90E-16
ko04510	Focal adhesion	128	1.61E-13
ko04151	PI3K-Akt signaling pathway	107	3.31E-13
ko04657	IL-17 signaling pathway	87	1.36E-09
ko04974	Protein digestion and absorption	88	8.50E-09
ko04320	Dorso-ventral axis formation	71	0.000364
ko04919	Thyroid hormone signaling pathway	78	0.004523
ko03022	Basal transcription factors	21	0.006703
ko04658	Th1 and Th2 cell differentiation	58	0.006703
ko04610	Complement and coagulation cascades	25	0.014555

Table S9. Significantly enriched KEGG pathways of DEGs in AS vs P group

Pathway ID	Pathway Name	Number of DEGs	Q value
ko04512	ECM-receptor interaction	223	3.82E-09
ko04151	PI3K-Akt signaling pathway	270	6.07E-07
ko03015	mRNA surveillance pathway	172	4.66E-06
ko04974	Protein digestion and absorption	234	9.24E-05
ko04510	Focal adhesion	315	0.000297
ko03013	RNA transport	246	0.000333
ko04657	IL-17 signaling pathway	217	0.000573
ko04260	Cardiac muscle contraction	76	0.001262
ko00330	Arginine and proline metabolism	60	0.001791
ko04919	Thyroid hormone signaling pathway	260	0.002528
ko04320	Dorso-ventral axis formation	211	0.011512
ko04261	Adrenergic signaling in cardiomyocytes	145	0.029084

Table S10. Significantly enriched KEGG pathways of DEGs in IS vs P group

Pathway ID	Pathway Name	Number of DEGs	Q value
ko03010	Ribosome	91	3.51E-20
ko04320	Dorso-ventral axis formation	101	2.19E-06
ko04658	Th1 and Th2 cell differentiation	90	3.31E-06
ko03022	Basal transcription factors	34	6.14E-06
ko04919	Thyroid hormone signaling pathway	114	1.53E-05
ko04330	Notch signaling pathway	104	2.63E-05
ko04151	PI3K-Akt signaling pathway	105	5.38E-05
ko04512	ECM-receptor interaction	178	0.001337
ko04510	Focal adhesion	120	0.001645
ko04974	Protein digestion and absorption	89	0.001974
ko03040	Spliceosome	93	0.008051

Table S11. Significantly enriched KEGG pathways of DEGs in HS vs P group

Pathway ID	Pathway Name	Number of DEGs	Q value
ko03010	Ribosome	220	1.07E-34
ko00190	Oxidative phosphorylation	103	4.49E-11
ko03022	Basal transcription factors	82	5.72E-09
ko03015	mRNA surveillance pathway	171	3.31E-08
ko04390	Hippo signaling pathway	92	1.78E-05
ko03040	Spliceosome	243	4.55E-05
ko03013	RNA transport	223	9.55E-05
ko04145	Phagosome	180	0.000137
ko04714	Thermogenesis	182	0.000193
ko04540	Gap junction	81	0.000467
ko04510	Focal adhesion	295	0.000695
ko04530	Tight junction	215	0.000877
ko04151	PI3K-Akt signaling pathway	229	0.001019
ko04919	Thyroid hormone signaling pathway	256	0.002332
ko04261	Adrenergic signaling in cardiomyocytes	138	0.005825
ko04512	ECM-receptor interaction	254	0.006765
ko04260	Cardiac muscle contraction	79	0.006951
ko04670	Leukocyte transendothelial migration	111	0.006951
ko04810	Regulation of actin cytoskeleton	154	0.007657
ko04966	Collecting duct acid secretion	41	0.030166
ko00010	Glycolysis / Gluconeogenesis	51	0.033021
ko04068	FoxO signaling pathway	76	0.045383
ko04722	Neurotrophin signaling pathway	151	0.049489

Table S12. Significantly enriched KEGG pathways of DEGs in MS vs P group

Pathway ID	Pathway Name	Number of DEGs	Q value
ko03010	Ribosome	220	5.37E-43
ko00190	Oxidative phosphorylation	103	2.82E-12
ko03022	Basal transcription factors	82	4.84E-11
ko03015	mRNA surveillance pathway	171	2.96E-06
ko03040	Spliceosome	260	4.12E-06
ko04714	Thermogenesis	176	1.29E-05
ko04145	Phagosome	189	5.63E-05
ko04260	Cardiac muscle contraction	79	0.000133
ko03013	RNA transport	241	0.000535
ko00010	Glycolysis / Gluconeogenesis	56	0.000889
ko04540	Gap junction	106	0.001853
ko04390	Hippo signaling pathway	96	0.001913
ko04512	ECM-receptor interaction	282	0.002337
ko04919	Thyroid hormone signaling pathway	335	0.002728
ko00830	Retinol metabolism	63	0.002865
ko04261	Adrenergic signaling in cardiomyocytes	155	0.007605
ko04530	Tight junction	292	0.008066
ko04510	Focal adhesion	400	0.009021
ko00980	Metabolism of xenobiotics by cytochrome P450	46	0.016778
ko04068	FoxO signaling pathway	94	0.019117
ko04670	Leukocyte transendothelial migration	111	0.019117
ko04964	Proximal tubule bicarbonate reclamation	24	0.019117
ko04151	PI3K-Akt signaling pathway	317	0.030231
ko00051	Fructose and mannose metabolism	30	0.030625
ko04320	Dorso-ventral axis formation	202	0.031506
ko04152	AMPK signaling pathway	102	0.033432
ko04666	Fc gamma R-mediated phagocytosis	62	0.044423
ko04810	Regulation of actin cytoskeleton	183	0.044888
ko01200	Carbon metabolism	94	0.045113

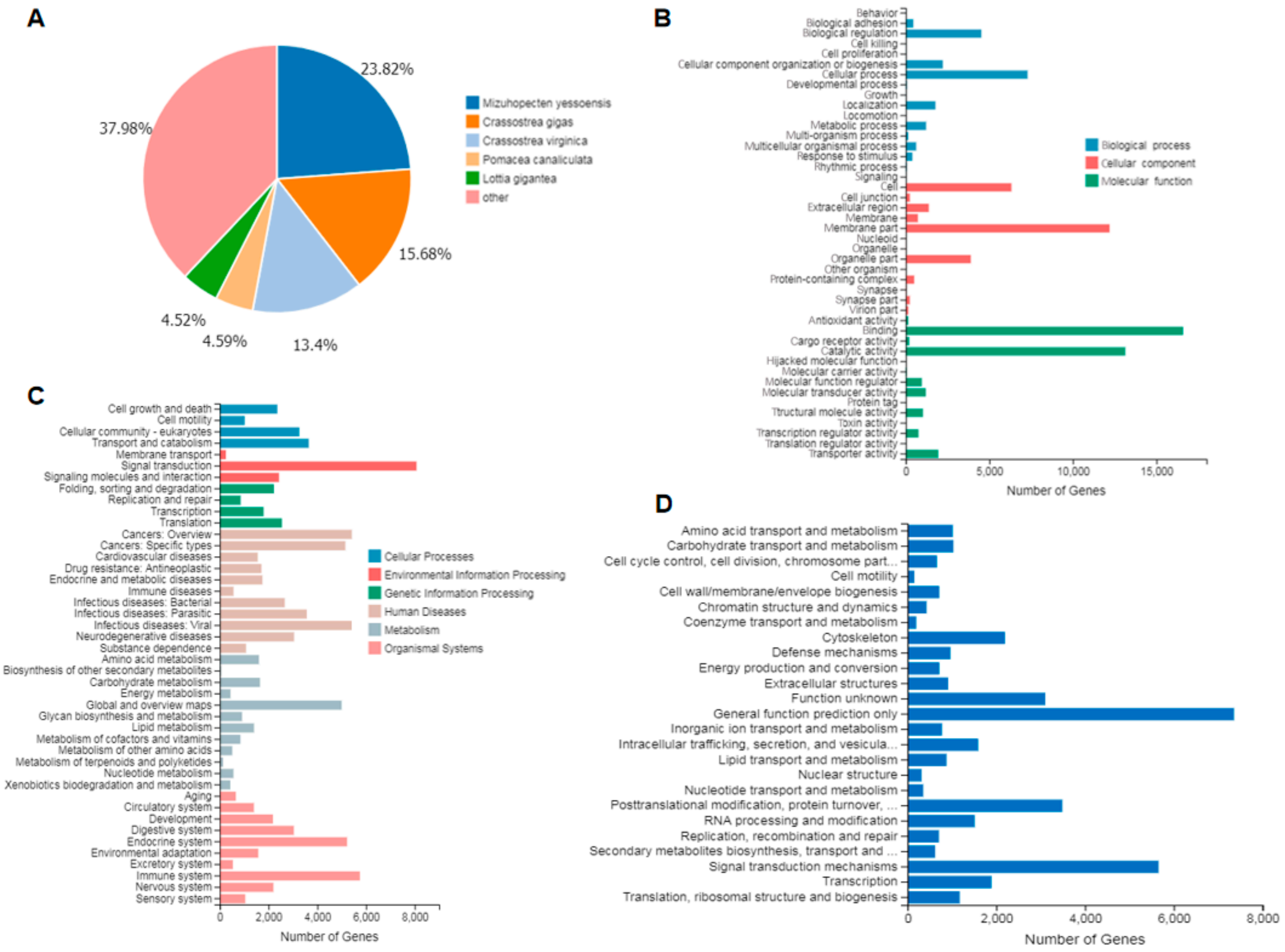

Figure S1. Unigene Classification. A: Species classification by NR annotation; B: GO classification; C: KEGG classification; D: KOG classification.
